# Analysis Model of Spoken English Evaluation Algorithm Based on Intelligent Algorithm of Internet of Things

**DOI:** 10.1155/2022/8469945

**Published:** 2022-03-27

**Authors:** Nan Xue

**Affiliations:** School of Foreign Languages, Xidian University, Xi'an, Shanxi 710071, China

## Abstract

With the in-depth promotion of the national strategy for the integration of artificial intelligence technology and entity development, speech recognition processing technology, as an important medium of human-computer interaction, has received extensive attention and motivated research in industry and academia. However, the existing accurate speech recognition products are based on massive data platform, which has the problems of slow response and security risk, which makes it difficult for the existing speech recognition products to meet the application requirements for timely translation of speech with high response time and network security requirements under the condition of network instability and insecurity. Based on this, this paper studies the analysis model of oral English evaluation algorithm based on Internet of things intelligent algorithm in speech recognition technology. Firstly, based on the automatic machine learning and lightweight learning strategy, a lightweight technology of automatic speech recognition depth neural network adapted to the edge computing power is proposed. Secondly, the quantitative evaluation of Internet of things intelligent classification algorithm and big data analysis in this system is described. In the evaluation, the evaluation method of oral English characteristics is adopted. At the same time, the Internet of things intelligent classification algorithm and big data analysis strategy are used to evaluate the accuracy of oral English. Finally, the experimental results show that the oral English feature recognition system based on Internet of things intelligent classification algorithm and big data analysis has the advantages of good reliability, high intelligence, and strong ability to resist subjective factors, which proves the advantages of Internet of things intelligent classification algorithm and big data analysis in English feature recognition.

## 1. Introduction

There are many research achievements in speech recognition, mainly including different speech recognition, feature extraction and recognition in complex environment, and real-time classification of a variety of speech [1]. In the conventional methods of speech recognition, voiceprint recognition and speech recognition are mostly used for multidimensional analysis, but the accuracy of this analysis method is greatly affected by environmental noise [2]. In recent years, in order to improve the accuracy of speech recognition, most scholars have improved speech recognition methods and data analysis strategies, including language recognition methods, database processing strategies, and so on [3]. However, there are some problems in speech feature recognition, such as low efficiency, large error, and inconsistent model [4]. In addition, the speech recognition technology and estimation algorithm adopted in different application scenarios are also quite different [5]. Based on this background, this paper proposes an oral English recognition method based on the integration of intelligent classification algorithm of Internet of things and big data analysis.

This paper studies the English text and English feature recognition system based on Internet of things intelligent classification algorithm and big data analysis, which is organized as follows.  Section 1 introduces the research background and overall framework of this study.  Section 2 introduces the research status of speech recognition and oral feature evaluation at home and abroad.  Section 3 adopts the intelligent classification algorithm of the Internet of things based on lightweight learning strategy, constructs the automatic speech and oral recognition model based on the intelligent classification algorithm of the Internet of things and big data analysis, and proposes the intelligent evaluation model of the effect of oral English recognition.  Section 4 verifies the reliability of the spoken English and English feature recognition system constructed in this paper, analyzes the experimental results and errors, and draws a conclusion.

Compared with the existing research results (the speech recognition method is only limited to the feature recognition of audio), the innovation of this paper is to propose the application of intelligent classification algorithm based on the Internet of things in speech recognition technology and the quantitative analysis of oral English evaluation algorithm. Through the daily recording and storage of different oral English data, make full use of the semantic differences between different oral English features and integrate the key data Information comparison and analysis to realize the closed-loop evaluation of oral English in the process of recognition.

## 2. Related Work

In recent years, a lot of research has been carried out on speech intelligent recognition, and some scientific research achievements have been made [6]. Calandruccio and other scholars improved the information input mode of the existing oral English evaluation model, proposed an English speech analysis model based on multidimensional genetic algorithm, used the multidimensional dispersion strategy to collect the data signals of the English feature recognition model, and realized the information normalization processing of the data signals through the Shenjing network algorithm [7]. Yazdani and other scholars put forward targeted evaluation strategies for the identification of different spoken English according to the differences in oral English expression [8]. Gupta and other scholars found that most speech recognition models still follow the traditional speech evaluation strategy and ignore the use of Internet of things technology and data twin technology. Therefore, the current oral English analysis models often adopt the characteristics of freeze frame input and propose a method of clustering analysis and processing of oral English data information based on characteristic regions, achieving high-quality evaluation of oral English recognition [9]. In order to improve the accuracy of speech recognition, Hovsepyan and other scholars proposed an innovative method of speech recognition based on neural network algorithm and Internet of things topology theory [10]. By studying and analyzing the semantic differences of spoken English in different scenarios, Gla and other scholars put forward a new “point-to-point” ladder evaluation system of spoken English and verified the effectiveness of the speech recognition system in the process of different spoken English recognition through practice [11]. Aiming at the problem of high error rate in speech recognition technology, Hülsmeier et al., combined with multivariate screening strategy and mesh analysis method, proposed an environment fusion recognition method based on Internet of things intelligent algorithm [12].

To sum up, it can be seen that in the process of recognizing spoken English, current speech recognition systems mostly use speech recognition technology to innovate in speech feature extraction, while ignoring its internal speech relevance and innovation [13, 14]. In addition, in terms of English feature recognition, although it can realize the local feature recognition of most spoken English, there is still no universal speech recognition intelligent model with high recognition accuracy [15–17]. On the other hand, most of the current research results focus on the speech recognition model with the intelligent analysis dimension as the core, and there are few speech recognition models combined with the intelligent algorithm of the Internet of things.

## 3. Methodology

### 3.1. Application of Internet of Things Intelligent Algorithm in the Construction of English Feature Recognition Model

**Figure 1:**
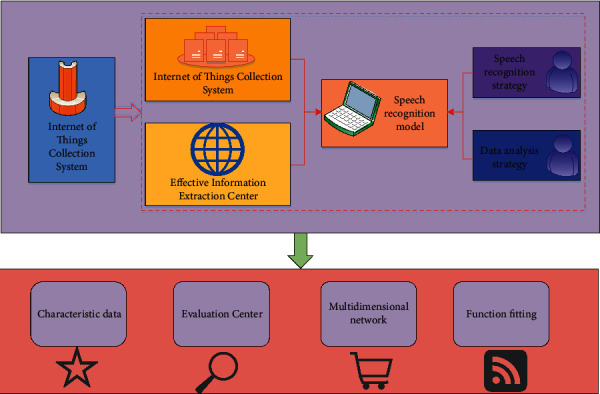
Theoretical methods and implementation processes often used in intelligent algorithms of the Internet of things.

In the application of speech recognition model, most speech recognition technologies are combined with quantitative analysis methods, audio acquisition methods, and information methods. In the early stage, their combined model was applied to audio recognition of information system [18]. In recent years, the intelligent classification algorithm and big data analysis strategy of the Internet of things have been fully applied in many industries, mainly to solve the identification of specific targets in specific scenarios and realize the efficient analysis and quantitative evaluation of environmental information by collecting environmental information and converting environmental information into characteristic data.  Figure 1 shows the commonly used theoretical methods and their implementation process [19]. The intelligent classification algorithm of the Internet of things has a similar idea of big data analysis and modeling, mainly by determining the mathematical relationship between many factors in different types of data, then extracting and classifying the differentiated features of the data, and then conducting intelligent analysis according to the known rules and matching strategies, so as to complete the multidimensional fusion of data [20]. In recent years, with the deepening of the research on intelligent classification algorithm and big data analysis of the Internet of things, its application in various fields is also gradually increasing, so speech recognition is starting to combine with more algorithms [21]. For example, in the fields of medicine, science and technology, transportation, agriculture, economy, and so on, it can be combined with the hardware sensors in the Internet of things to complete the collaborative processing and quantitative analysis of data in different dimensions [22].

Based on the intelligent algorithm of Internet of things, combined with the main characteristics of spoken English, relying on speech recognition technology and spoken language database, the quantitative evaluation of spoken English can be realized by adopting the process of “audio collection-noise removal-feature extraction-text recognition-semantic extraction.” Based on this, the construction process of this intelligent model is as follows.

The first step is to measure the degree of relevance according to the relationship or similarity between oral English and environment, mainly by combining the speech recognition strategy model to realize the feedback analysis of its accuracy. In the application of this model, the original spoken English data matrix is initialized, and then the reference data column is formulated and characterized by data. As shown in formula (1), the demand condition on one side is expressed, the actual value of the individual corresponding to the other side is *Q*_*erca*_, the average value of all individuals on the other side is *Q*_*erca*_′ the maximum value corresponding to the largest individual is *Q*_max_, and the minimum value corresponding to the smallest individual is *Q*_min_.

(1) $$\begin{array}{c} M\left(x\right)=Q_{erca}^{e-1}{\left(\frac{Q_{erca}+Q_{\min}}{Q_{\max}-Q_{\min}}\right)}^{x}. \end{array}$$

In the feature recognition formula (formula (1)) of spoken English, the calculation condition of evaluation accuracy is (*Q*_*erca*_ − *Q*_min_/*Q*_min_) ≤ 1 and the expression of accuracy *Q*′(*x*) is

(2) $$\begin{array}{c} Q^{\prime }\left(x\right)=\frac{Q^{e-1}{}_{erca}}{x/x-1}{\left(\frac{Q_{erca}+Q_{\min}}{Q_{\max}-Q_{\min}}\right)}^{x}. \end{array}$$

The English feature recognition accuracy formula (formula (2)) shows that when the satisfaction calculation condition is (*Q*_*erca*_ − *Q*_min_/*Q*_min_) ≥ 1, the coincidence expression *W*(*x*) of the corresponding oral English evaluation algorithm is

(3) $$\begin{array}{c} W\left(x\right)=\frac{{\left(Q_{erca}+Q_{\min}/Q_{\max}-Q_{\min}\right)}^{ex}\left(x-de^{x}/x+1\right)}{e^{x}-1}, \end{array}$$

where *d* is the environmental factor and *e*^*x*^ is the time factor.

### 3.2. Construction of Oral English Evaluation Function in Big Data Analysis Strategy Based on Intelligent Classification Algorithm of Internet of Things

**Figure 2:**
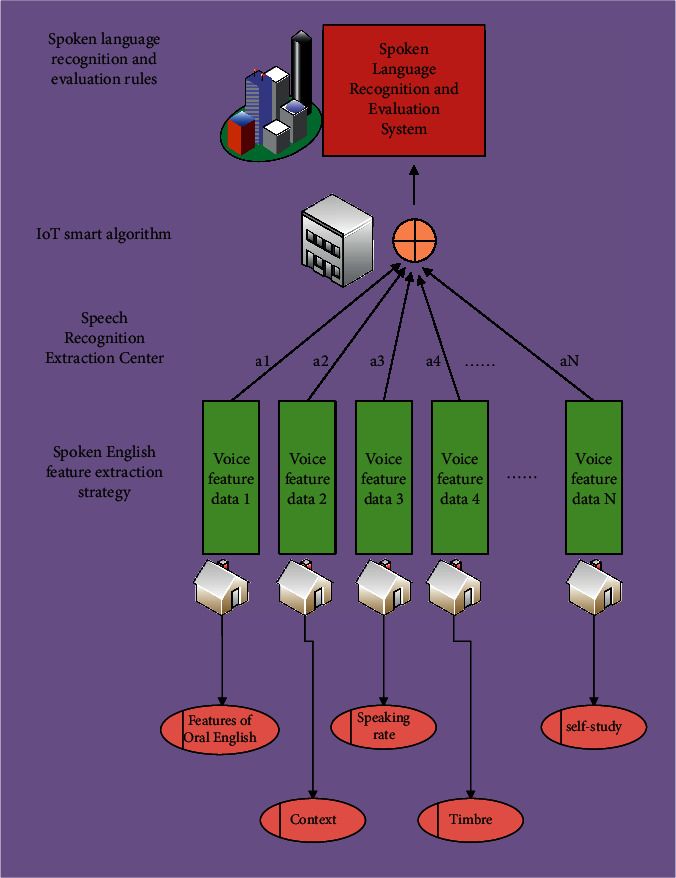
The evaluation process of spoken English data.

After the coupling analysis of oral English by using the intelligent classification algorithm of the Internet of things, its internal relevance and direct data coupling have a good dimension. Oral test directly detects the examinee's language performance, with strong subjectivity and low reliability. This paper discusses the feasibility of constructing a scientific oral evaluation system from the quality indicators of reliability and validity, combined with the oral examination links such as test construct, information feed and output, and examination form and score. Therefore, it is also necessary to construct the oral English evaluation function on the basis of the above research. The construction principle is the internal relevance analysis strategy of data, and its evaluation process of data is shown in Figure 2.

**Figure 3:**
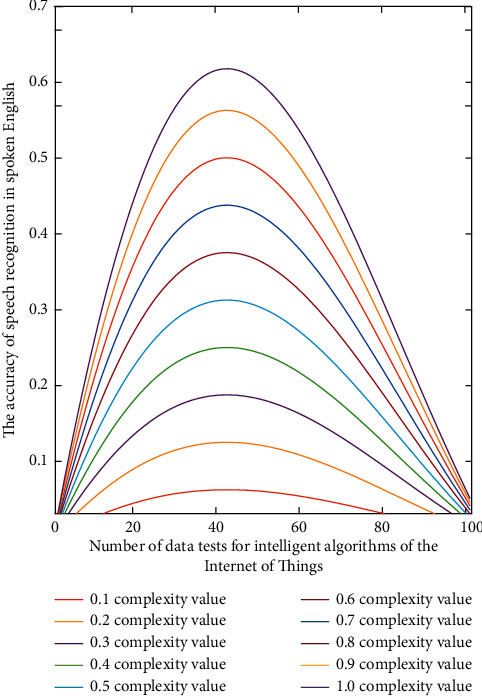
Analysis of the accuracy of the speech recognition of spoken English in different complexities of the intelligent algorithm of the Internet of things.

In the above oral English evaluation model, we can judge the accuracy of oral English through different dimensions. Although this evaluation method can reduce the interference of other factors and objectively reflect the difference of influence, there will be a certain degree of misjudgment in the evaluation of spoken English, resulting in inaccurate final evaluation results. If the low probability misjudgment factors in the process of speech recognition are not considered, the solution value of the objective function is the smallest. It shows that the voice evaluation function value is the best, and the data analysis and classification process are shown in Figure 3.

As can be seen from Figure 3, with the increase of oral complexity in 10 groups of data, its internal relevance and accuracy have improved, especially in the evaluation of oral English in complex environment. If the vector of the second matrix is *H*_*i*_, the single extreme value can be set to *F*_*i*_, and the set extreme value can be set to *T*_*i*_. The relationship between one-dimensional equation and two-dimensional equation in algorithm calculation is as follows:

(4) $$\begin{array}{c} T_{i}=\frac{\lambda^{2}}{\lambda^{2}+2}\sqrt{\frac{H_{i}+F_{i}}{\lambda }},\\ T_{i}^{\prime }=\frac{\lambda^{2}}{\lambda^{2}+2}\sqrt{\frac{H_{i}^{\prime }+F_{i}^{\prime }}{\lambda }}+\frac{\sqrt{x-w^{2}\lambda /x+1}}{\lambda }, \end{array}$$

where *w* is the multidimensional weight of the evaluation function. In order to further remove the noise factors in the environment, the calculation formula can be expressed as

(5) $$\begin{array}{c} T_{i}=\frac{\left(\lambda^{2}/\lambda^{2}+2\right)\sqrt{H_{i}+F_{i}/\lambda }}{H_{i}^{2}+F_{i}^{2}},\\ T_{i}^{\prime }=\frac{\left(\lambda^{2}/\lambda^{2}+2\right)\sqrt{H_{i}^{\prime }+F_{i}^{\prime }/\lambda }+\left(\sqrt{x-w^{2}\lambda /x+1}/\lambda \right)}{H_{i}^{2}+F_{i}^{2}}. \end{array}$$

**Figure 4:**
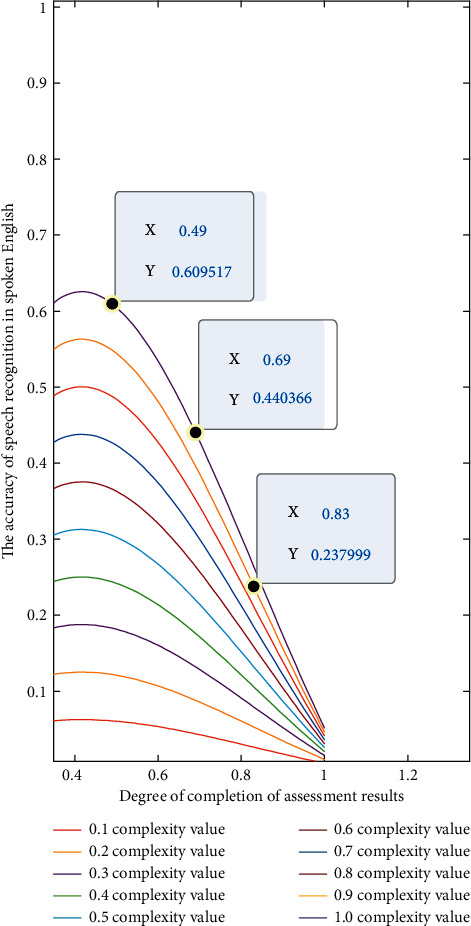
Calculation flowchart for detecting changes in English text.

The calculated accuracy results under different evaluation degrees are shown in Figure 4.

It can be seen from the evaluation results in Figure 4 that under different degrees of oral complexity, there are important differences in the internal relevance and data consistency in the process of language recognition technology. For example, when the evaluation completion degrees are 0.49, 0.69, and 0.83, the difference in the corresponding accuracy is very obvious, basically showing the law of equal difference sequence. This is because the optimization method based on the intelligent algorithm of the Internet of things mainly makes cyclic iterative judgment by setting the threshold. When the cyclic result meets the set threshold requirements, the corresponding final result can be output. In this experiment, the program is written in MATLAB language. The iterative automatic threshold algorithm is used to realize the global threshold segmentation and local threshold segmentation of the intelligent algorithm. Using the results of global threshold segmentation, the edge detection of the picture is realized, and the data information in the algorithm is enhanced.

**Figure 5:**
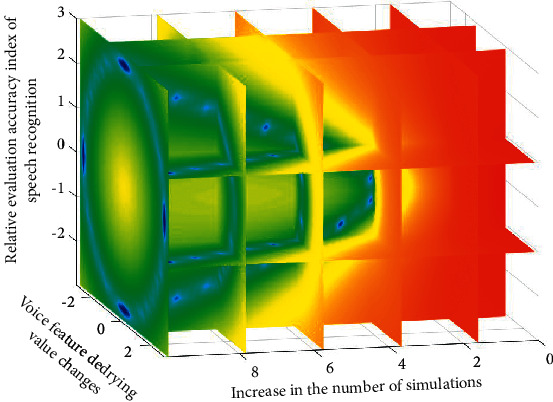
Analysis of 3D simulation results under the intelligent algorithm of the Internet of things.

The optimization model of speech recognition based on intelligent algorithm of Internet of things adopts the optimization idea of greedy iterative algorithm. Compared with the conventional quantitative evaluation method, this iterative method can have higher reliability and smaller evaluation error with the increase of evaluation times. However, after optimization, the objectivity of the evaluation can be greatly changed. Therefore, in this optimized evaluation method, through three-dimensional simulation verification, the three-dimensional simulation results are shown in Figure 5.

As can be seen from Figure 5, with the increase of simulation times, different types of speech samples can quickly characterize their internal relationship under the same denoising conditions, which is also in line with the expected simulation results and simulation rules. In the evaluation process of simulation results, the red color represents the worse evaluation results, and the blue color represents the better evaluation results. Assuming that the factor universe is represented by discrete function and the evaluation level universe is represented by time value, the vector expressions of the evaluation function before denoising *S*_*i*_(*x*) and after denoising *S*_*i*_′(*x*) are, respectively,

(6) $$\begin{array}{c} S_{i}\left(x\right)=\frac{\sqrt{1-xe^{x}/x+e^{x}}}{xe^{x}+2e^{x-1}},\\ S_{i}^{\prime }\left(x\right)=\frac{\sqrt{xe^{1-x}+e^{x}/1+e^{x}}}{x-2+xe^{x-1}}. \end{array}$$

### 3.3. Simulation Solution Process of English Text and English Feature Recognition Model

**Figure 6:**
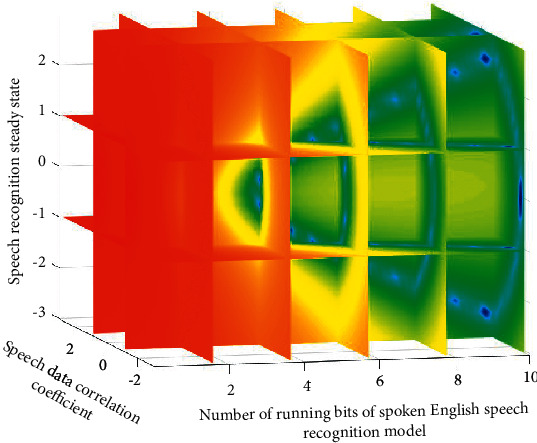
Three-dimensional simulation results of data corresponding to the English feature recognition model.

After analyzing and summarizing the characteristics of spoken English, it is necessary to calculate the relationship or correlation degree of different simulation data columns with the help of its internal correlation and the type of spoken English evaluation strategy and then sort the correlation degree. The three-dimensional simulation results of the data corresponding to the English feature recognition model in this link are shown in Figure 6.

It can be seen from the results in Figure 6 that in the process of simulation analysis of different data types, a variety of data gradually change from unstable to stable, and the result orientation of their recognition effect is also significantly different because different data types contain different English features.

In the process of identifying spoken English, the meaning of spoken English in different environments is also different, so equivalent analysis cannot be carried out, and the original data need to be processed dimensionless. After calculating the absolute difference between each factor and the main factor at the same observation point, it is also necessary to calculate the correlation degree between each subfactor and the main factor in the simulation process. When comprehensively evaluating things, the problem of ranking will be involved in most cases. Each evaluation object needs to be ranked first, so grey comprehensive evaluation is also required. The formula is

(7) $$\begin{array}{c} T\left(x\right)=\frac{\sqrt{1+e^{1-x}/1-e^{1+x}}+\sqrt{e^{1-x}/1+xe^{x}}}{\sqrt{1+xe^{x}}}. \end{array}$$

The optimized function expression is

(8) $$\begin{array}{c} T^{\prime }\left(x\right)=\frac{\sqrt{1+e^{1-x}/1-e^{1+x}}-\sqrt{e^{1-x}/1+xe^{x}}}{1+\sqrt{x+xe^{x}}}. \end{array}$$

Combined with the intelligent algorithm based on the Internet of things, use the improved algorithm and data analysis strategy proposed in this study to determine the weight of each index, improve the accuracy of index weight, and ensure that the weight distribution is more real. Select a reasonable evaluation level. The evaluation coupling function *R*(*x*) is

(9) $$\begin{array}{c} R\left(x\right)=x\sqrt{\frac{1}{x+xe^{x}}}. \end{array}$$

The optimized coupling function is

(10) $$\begin{array}{c} R^{\prime }\left(x\right)=\frac{x+e^{x}}{3xe^{x-1}}\sqrt{\frac{e^{x}-1}{x+xe^{x}}}. \end{array}$$

After calculating the coupling degree, the solution result *D*(*x*) can be expressed as

(11) $$\begin{array}{c} D\left(x\right)=\sqrt{\frac{R\left(x\right)+T\left(x\right)+S_{i}\left(x\right)}{xe^{x}}}. \end{array}$$

## 4. Experimental Design and Analysis Process of English Feature Recognition Model

### 4.1. Experimental Design

After processing based on the intelligent algorithm of the Internet of things, the feature information in oral English can be clustered, so that the corresponding noise in the oral expression environment can be effectively reduced, so as to better improve the recognition rate and accuracy of oral English. Then, the data information obtained by the self-learning machine learning algorithm is convoluted, and its internal correlation analysis is realized through decoupling analysis. During the experiment, many types of oral English samples are required (for example, British English and American English) which are input into the Internet of things for machine learning and feature extraction, and then the three features are fused. After the fused features are obtained, the convolution network and intelligent algorithm of the Internet of things are further used for feature extraction and processing, so as to obtain the final output of spoken English recognition results.

**Figure 7:**
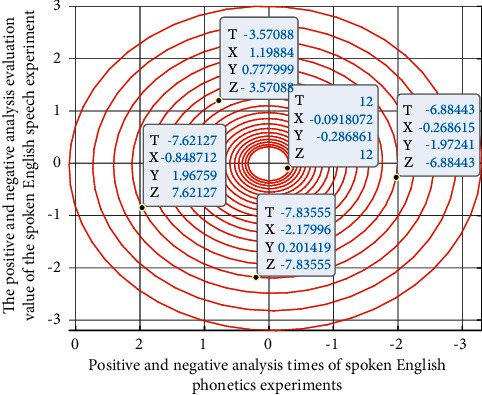
Scoring test results of spoken English assessment algorithm.

Before the formal experiment of the recognition model, it is necessary to determine the characteristic parameters of spoken English. According to the experimental samples, the recognition and evaluation rules of oral English are determined, and the characteristic parameters of English features are selected. The experimental process is shown in Figure 7. The tested objects are intelligently classified through the Internet of things. A total of no less than 1000 types of repeated screening and classification are carried out, and the average classification results are used as the preliminary experimental reference index.

### 4.2. Experimental Data Processing and Result Analysis

Figure 7 shows the score detection results of the spoken English evaluation algorithm. The relevant data in the speech feature recognition score detection model are analyzed and processed by MATLAB software and Python compiler.

As can be seen from the evaluation results in Figure 7, under different evaluation conditions (*t* data), with the increase of the number of experiments, the evaluation accuracy of the first single oral English experiment data (*x* data) will be improved to a certain extent, but for two-dimensional (*y* data) and three-dimensional (*D* data) data, with the increase of the number of experiments, the evaluation results also show a relatively stable trend (gathered in a certain range), so it has good stability, which also shows that the evaluation index of the identification system has high reliability and stability. Therefore, it can be seen from the experimental results that different types of data fluctuate greatly in the evaluation accuracy because different types of English speakers are different in the process of multiple screening and classification of experimental data. The stability and timbre characteristics of language data groups are different, and the English features involved in different data groups are different, and the initial feature parameter screening will also fluctuate. However, with the increase of the number of experiments, its volatility will weaken and its stability will enhance, so the accuracy of its evaluation will be higher and higher.

In addition, the evaluation index system of the oral evaluation system of the speech recognition technology based on the intelligent algorithm of the Internet of things refers to the speech recognition evaluation standard, which is divided into three levels, of which there are four secondary indicators, namely, the recognition score of the oral English evaluation algorithm, the oral meaning score, the accuracy score of oral feature recognition, and the discrimination score of oral regional features. Each evaluation index of spoken English is divided into different three-level indicators. The English feature recognition score has five three-level indicators, which can comprehensively reflect the recognition accuracy of spoken English.

## 5. Conclusion

This paper studies the application of intelligent classification algorithm based on Internet of things in the analysis of oral English evaluation algorithm based on speech recognition technology. The experimental results show that the spoken English feature recognition system based on Internet of things intelligent classification algorithm and big data analysis has the advantages of good reliability, high intelligence, and strong ability to resist subjective factors. It proves the advantages of Internet of things intelligent classification algorithm and big data analysis in English feature recognition. This paper solves the problems of large proportion of subjective factors and low degree of intelligence in English text and English feature recognition methods. However, this study only considers the processing of oral feature signals but does not consider the elimination of noise. The future research work can be deeply studied from two aspects: homologous audio separation and error evaluation methods.

## Data Availability

The experimental data used to support the findings of this study are available from the corresponding author upon request.
